# Case Report: Portable handheld ultrasound facilitates intra-articular injections in articular foot pathologies

**DOI:** 10.3389/fpain.2024.1254216

**Published:** 2024-02-29

**Authors:** Samir Ghandour, Atta Taseh, Walter Sussman, Daniel Guss, Soheil Ashkani-Esfahani, Ashim Gupta, Gregory Waryasz

**Affiliations:** ^1^ Foot & Ankle Research and Innovation Laboratory (FARIL), Department of Orthopedic Surgery, Massachusetts General Hospital, Harvard Medical School, Boston, MA, United States; ^2^ Good Samaritan Medical Center, Boston Sports & Biologics PC, Wellesley, MA, United States; ^3^ South Texas Orthopaedic Research Institute (STORI Inc.), Laredo, TX, United States; ^4^ Future Biologics, Lawrenceville, GA, United States; ^5^ BioIntegrate, Lawrenceville, GA, United States; ^6^ Regenerative Orthopaedics, Noida, India

**Keywords:** metatarsophalangeal joint, tarsometatarsal joint, intra-articular injections, portable ultrasonography, foot and ankle, sports medicine

## Abstract

**Background:**

Intra-articular injections are commonly used to manage joint pathologies, including osteoarthritis. While conventional ultrasound (US) guidance has generally improved intra-articular injection accuracy, forefoot and midfoot joint interventions are still often performed without imaging guidance. This pilot study aims to evaluate the efficacy of office-based, portable ultrasound (P-US) guided intra-articular injections for forefoot and midfoot joint pain caused by various degenerative pathologies.

**Methods:**

A retrospective analysis was conducted on a series of consecutive patients who underwent P-US guided intra-articular injections following a chief complaint of forefoot or midfoot joint pain. Patients reported their pain levels using the Visual Analog Scale (VAS) pre-injection and at 3 months follow-up. The procedure was performed by an experienced foot and ankle surgeon using a linear array transducer for guidance, and a 25-gauge needle was used to inject a combination of 2 cc 1% lidocaine and 12 cc of Kenalog (40 mg/ml). Complications and pain scores were analyzed using a paired *t*-test and *p* < 0.05 was considered significant.

**Results:**

We included 16 patients, 31% male and 69% female with a mean age (±SD) of 61.31 (±12.04) years. None of the patients experienced immediate complications following the intervention. The mean pre-injection VAS score was significantly reduced from 5.21 (±2.04) to a mean of 0.50 (±1.32) at 3 months follow-up (*P* < 0.001). Thirteen patients reported complete resolution of pain at the 3-month follow-up. No adverse events were reported throughout the duration of the study.

**Conclusion:**

This pilot study suggests P-US-guided intra-articular injections offer a safe and effective method for managing forefoot and midfoot joint pain caused by various arthritic pathologies. Further research is warranted to establish the long-term efficacy and comparative effectiveness of P-US-guided injections in larger patient cohorts as compared to non-image guided injections.

## 1 Introduction

With adequate training, care providers can manage various joint pathologies safely by targeted injections (1). Intra-articular injection procedures necessitate minimal setting requirements and may be performed in locations ranging from primary care clinics to field or training room clinics with proper sterile technique (1). The most common indications for intra-articular injections include joint pain or an arthrogram where a contrast agent is injected into the joint space (2, 3).

US guided injections have demonstrated increased accuracy regardless of target site (4). Moreover, the higher accuracy of US guided injections in the midfoot area has been well demonstrated by Khosla et al. (5). Despite this, interventions of the forefoot and midfoot joints are still routinely performed under palpation guidance relying on anatomic landmarks (6), which is considered less accurate and leads to complications such as extravasation or local inflammatory reactions (1, 7, 8). A study by Kraus et al. found that patients with pathological conditions like hallux valgus (HV) or 1st metatarsophalangeal (MTP) arthritis had higher rates of extra-articular injections when guided by palpation. This underscores the need for more accurate methods of guiding intra-articular injections in patients with articular pathology (9).

Office-based P-US guided procedures have become increasingly available to guide joint and soft tissue injections. Hand-held P-US devices are low-cost, easy to operate, and portable choices that offer acceptable accuracy comparable to that of conventional US (10). In this pilot study, we aim to illustrate the use and potential efficacy of office-based, P-US guided intra-articular injections for forefoot and midfoot joint pain caused by several arthritic and degenerative pathologies.

## 2 Methods

We retrospectively reviewed the charts of a consecutive series of 16 consecutive patients above the age of 18 years old, presenting to an orthopedic clinic at an academic tertiary setting for pain associated with forefoot or midfoot articular pathologies with a hallmark of arthritis or degenerative changes. This study was approved by the Foot and Ankle Clinical Database (IRB protocol #2015P000464) at Massachusetts General Hospital. Patients with first MTP or tarsometatarsal (TMT) joint arthritis were considered for inclusion. Patients with prior intra-articular injection of corticosteroids into the target joint were excluded. Everyone reported their pain using the Visual Analog Scale (VAS) prior to the procedure and at 3 months follow-up. They were monitored following the procedure for immediate complications such as injection material extravasation or limited joint range of motion. They were also asked to report any delayed complications during the subsequent 3 months after the injection. Possible delayed complications include post-injection flare, skin reaction, and infection as observed in a previous study involving US-guided intra-articular injections of other foot and ankle joints (11).

**Figure 1:**
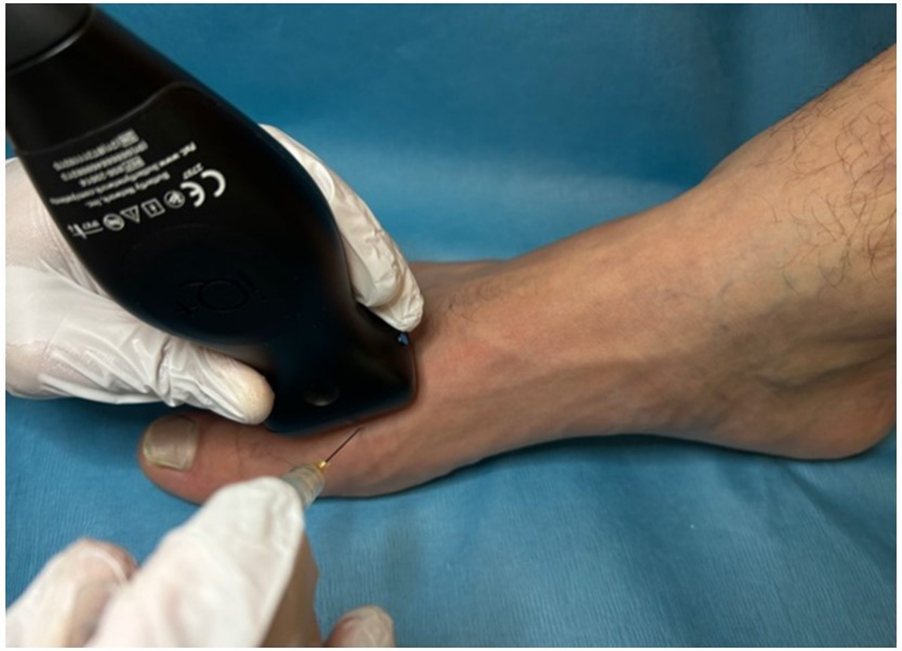
Out-of-plane approach for intra-articular injection of the first metatarsophalangeal joint.

**Figure 2:**
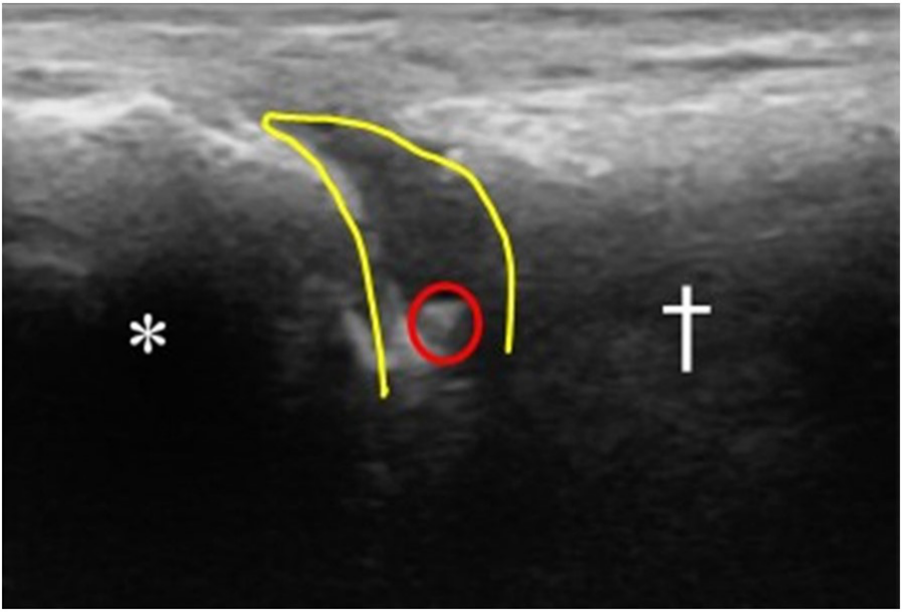
Sagittal view under P-US of the 1st MTP joint after injection. (*) marks the proximal phalanx. (†) marks the distal end of the 1st metatarsal bone. The tip of the needle is seen within the joint space circled in red. The joint space is outlined in yellow, showing the distension of the space following injection (anechoic space). This indicates successful injection of the material intra-articularly.

Prior to the injection, the possible complications of steroid injections were reviewed with the patient and informed consent was obtained. The patient was then positioned supine with the knee bent at 90° and the foot neutral and positioned on the table. The injection site was prepared with Ethyl Chloride Topical Anesthetic Spray and then cleaned using a ChloraPrep swab stick with 2% chlorhexidine gluconate and 70% isopropyl alcohol. A linear array transducer (Butterfly iQ+™ P-US probe 1–10 MHz, Burlington, MA, USA) was used to scan the dorsal forefoot along the sagittal plane and to confirm the symptomatic joint. Using an out-of-plane approach, a 1.5 cm 25-gauge needle (8) was advanced into the joint space under direct visualization, aspirating the joint to confirm intra-articular access (Figure 1). Still under direct visualization, the joint was injected with 2 cc of 1% Lidocaine and 1 cc of Kenalog (40 mg/ml) into the articular joint space (Figure 2). Following the injection, the access point was cleaned using an alcohol swab and the patient is observed for any immediate complications.

A paired *t*-test using SPSS 28 (IBM Corp., Armonk, New York, USA) was performed for statistical analysis. *P* < 0.05 was considered significant.

## 3 Results

**Figure 3:**
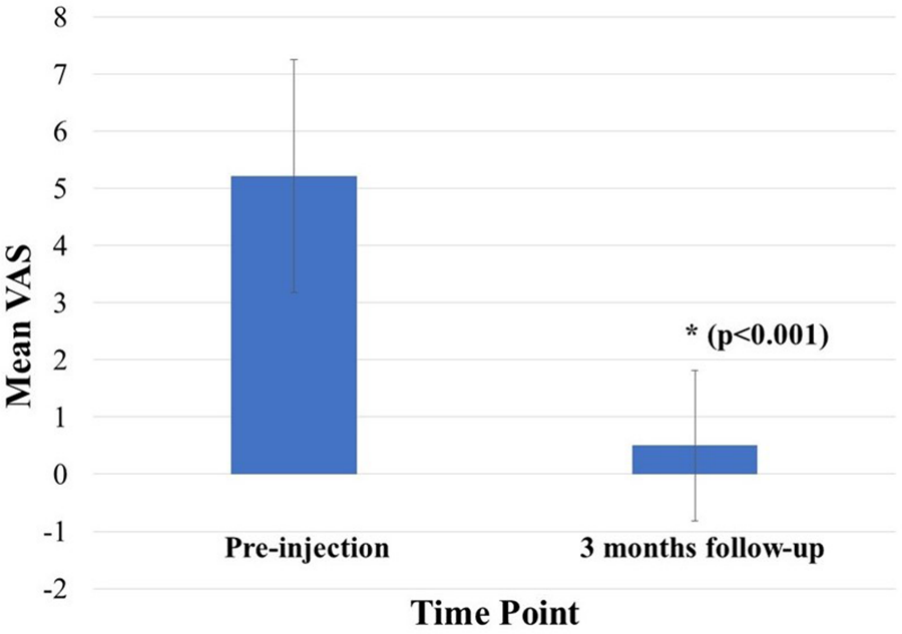
Pre- and post-injection (3 months follow-up) mean VAS scores. * shows *p* < 0.001.

Sixteen patients (31% male; 69% female) with a mean age of 61.31 (±12.04) years, underwent P-US guided injections. Thirteen injections were done in the first MTP joint, and three were done in the TMT joints. None experienced immediate complications following the intervention or during the 3-month period following the injection. The mean (±SD) reported pre- and post-injection (3 months follow-up) VAS pain scores were 5.21 (±2.04) and 0.50 (±1.32), respectively (Table 1). The difference between both related outcome scores was statistically significant (*p* < 0.001). Thirteen of the subjects reported complete resolution of pain on subsequent follow-up at 3 months. Figure 3 illustrates the mean pre-injection and post-injection VAS pain scores.

**Table 1:** Patients’ diagnosis, VAS pain scores, and procedural complications.

Patient number	Diagnosis	Pre-injection VAS	Post-injection VAS (3-months)	Immediate or delayed complications
1	Hallux rigidus	6	0	–
2	2nd TMT arthritis	7	5	–
3	Hallux rigidus with mild medial eminence tenderness	5	0	–
4	Hallux limitus	5	0	–
5	Hallux limitus	8	0	–
6	4th TMT joint arthritis/synovitis	6	2	–
7	Hallux limitus	4	1	–
8	Hallux rigidus	4	0	–
9	Hallux rigidus	7	0	–
10	Hallux rigidus	8	0	–
11	Hallux rigidus	5.5	0	–
12	2nd TMT arthritis	5	0	–
13	Hallux rigidus	5	0	–
14	Hallux rigidus	6	0	–
15	Hallux rigidus	1	0	–
16	Hallux rigidus	1	0	–

## 4 Discussion

This case series reviewed the clinical outcomes of 16 patients with a variety of midfoot and forefoot pathologies who underwent P-US guided intra-articular injections for the short-term management of articular joint pain. On subsequent follow-up, all patients showed significant improvement in pain on the VAS pain score, with most (13/16) reporting complete resolution of pain at the 3 months follow-up. There were no immediate or delayed complications. These findings highlight the safety and efficacy of handheld P-US guided corticosteroid injections for the short-term management of articular joint pain.

Previously published studies have likewise shown high accuracy and safety with US-guided injections in the upper and lower extremity. When compared to landmark-guided injections, US-guided injections have been superior with an accuracy of 100% in each study compared to 58%–85% in those with palpation guidance (12–17). However, fewer studies have examined if this translates to improved efficacy, especially in smaller foot joints and under P-US guidance. In a cadaveric study, Khosla et al., reported 100% accuracy for both US and palpation guided injections in subtalar and ankle joint injections (5). Although, US guidance significantly improved midfoot injections in their study (*p* = 0.003), fluoroscopy was still superior in accuracy. Interestingly, out of the three cases who did not experience complete resolution of pain in our study, two of them had injections in their midfoot joints. Jha et al., did the same comparison for talonavicular joint injections. Out of five palpation-guided injections, four of them were placed extra-articularly and one in the wrong joint (naviculocuneiform). On the contrary, all five injections in the US group were placed correctly (18).

Clinical outcomes of the injections were also investigated by some authors. Anderson et al., retrospectively reviewed 1,708 patients' charts who had intra-articular injections in their ankle or subtalar joints. They reported a 5.8% rate of adverse events within 90 days after the injection, with the most common one being the post-injection flare (11). In a clinical trial, Razavi et al., compared palpation-guided (*n* = 25) vs. ultrasound-guided (*n* = 25) injections in hallux rigidus patients. They did not find any differences in terms of symptomatic relief in their 2- and 6-weeks follow-up visits. They also did not find any complications in either group (19). In another study comparing the accuracy of palpation and US-guided intra-articular needle placement in the small joints of the hand (proximal interphalangeal and metacarpophalangeal joints), Raza et al. showed that 59% of palpation-guided injections were accurate compared to 96% in the US-guided injections group (13). They observed no complications in those who underwent US-guided injections. The literature is lacking on the clinical outcomes of the injections in the midfoot area. Since these joints seem to be the most challenging, future studies should assess the clinical efficacy of P-US for injections in this area.

Comparing the efficacy and the incidence of complications in our patient series to the literature reported on palpation-guided injections shows a potential superiority and reliability of P-US guided intra-articular injections (7, 8, 20). P-US has become widely used in musculoskeletal medicine to assist with guided injections. P-US guidance allows the clinician to visualize adjacent anatomic structures, minimize risk to adjacent structures, and increase the accuracy over traditional landmark-guided injections. It also avoids exposing patients to radiation associated with fluoroscopy, and office-based P-US may be performed in the clinic at a decreased cost given that the probe is a one-time purchase with no operating costs and without the safety requirements of lead-lining an exam room (21, 22). Moreover, its use avoids the logistical and financial burden of receiving injection guidance using conventional US which is only available at specialized centers and radiology departments. In a randomized controlled trial by Sibbit et al., the authors found that the use of conventional US-guidance was 8% more cost-effective and had a 32% longer duration of pain reduction when compared to palpation-guided injections (23). Considering the degree of technical advancement of US technology since the time of this trial in 2009, P-US should perform at least as well as US when compared to palpation guidance. Combined, these advantages may provide not only an opportunity to improve the accuracy of intra-articular injections, but also a cost-effective alternative when compared to other guidance modalities, or lack thereof.

With the emergence of handheld P-US, there is little to no evidence in the literature on their efficacy compared to that of conventional US. Conventional US machines are naturally more robust as they are more technically sophisticated when compared to the simplified hardware of P-US. This translates to lower resolution and capabilities in P-US, which may decrease trust among providers for its use in applications readily established for conventional US. Studies like this show that despite their hardware limitations, P-US probes are non-inferior to conventional US probes when it comes to specific applications—in this case, guided injections.

## 5 Limitations

An important limitation of this case series is the small sample size (*n* = 16). In addition, the three-month follow-up period was not designed to assess for recurrence of pain and the need for subsequent re-injection (24). We also did not recruit a control group of palpation-guided or conventional US-guided injections for comparative purposes to formally evaluate the relative value of using P-US for guidance, as we perform all intra-articular injections under P-US at our clinic. Another limitation is that all injections were performed by a single foot and ankle orthopedic surgeon. Finally, the pathologies we treated with intra-articular injections were not objectively verified, as they were clinically diagnosed by the treating surgeon following a thorough physical and history examination correlated with radiographic findings. Given that ultrasonography is operator-dependent, additional studies are necessary with a broader group of providers.

## 6 Conclusion

We have demonstrated that P-US guided intra-articular injections for forefoot and midfoot joint pain caused by various arthritic pathologies offer potential advantages over palpation-guided injections, including improved accuracy and few if any complications. Future comparative studies with larger sample sizes, longer follow-up periods, and matched control groups are needed to validate our findings. Nonetheless, this study provides valuable insight into the potential benefits of P-US guided intra-articular injections for forefoot and midfoot joint pain which warrants further investigation.

## Highlights

• The study shows the potential advantages of portable ultrasound (P-US) guided intra-articular injections for managing forefoot and midfoot joint pain caused by various arthritic pathologies. Compared to palpation-guided injections, P-US guided injections offer improved accuracy and fewer complications.
• The study, performed in a clinical setting, used a P-US device to guide joint and soft tissue injections. Sixteen patients with midfoot and forefoot articular pathologies were administered these injections and their outcomes were monitored.
• No immediate or delayed complications were observed post-injection in any of the patients, highlighting the safety of the procedure when P-US is used for guidance. Most patients (13 out of 16) reported complete resolution of pain at the 3-month follow-up, showing the efficacy of P-US guided injections.
• The study also emphasizes the benefits of P-US guidance including increased accuracy over traditional landmark-guided injections, no exposure to radiation, and decreased costs due to the one-time purchase of the probe and lack of operating costs.
• Despite the encouraging results, the study's limitations include its small sample size, the absence of a control group, and the fact that all injections were performed by a single orthopedic surgeon. Therefore, further studies are needed with larger sample sizes, longer follow-up periods, and a broader group of providers to validate these findings.

## Data Availability

The original contributions presented in the study are included in the article/Supplementary Material, further inquiries can be directed to the corresponding author.

## References

[1] Wittich CM Ficalora RD Mason TG Beckman TJ. Musculoskeletal injection. Mayo Clin Proc. (2009) 84(9):831–7. 10.4065/84.9.831 19720781 PMC2735433

[2] Balog TP Rhodehouse BB Turner EK Slevin JM Bush LA Grassbaugh JA Accuracy of ultrasound-guided intra-articular hip injections performed in the orthopedic clinic. Orthopedics. (2017) 40(2):96–100. 10.3928/01477447-20161213-03 27992639

[3] Patel A Chadwick N von Beck K Goswami P Soliman SB Patel A Ultrasound-guided joint interventions of the lower extremity. Skeletal Radiol. (2023) 52(5):911–21. 10.1007/s00256-022-04168-5 36042035

[4] Daniels EW Cole D Jacobs B Phillips SF. Existing evidence on ultrasound-guided injections in sports medicine. Orthop J Sports Med. (2018) 6(2):2325967118756576. 10.1177/2325967118756576 29511701 PMC5826008

[5] Khosla S Thiele R Baumhauer JF. Ultrasound guidance for intra-articular injections of the foot and ankle. Foot Ankle Int. (2009) 30(9):886–90. 10.3113/FAI.2009.0886 19755074

[6] Reilly I Bromley G Flanagan G. *A systematic review of injectable corticosteroid for osteoarthritis of the first metatarsophalangeal joint**.* *In Review* (2020). Available online at: https://www.researchsquare.com/article/rs-105785/v1 (Accessed 2023 Jun 12).

[7] Leopold SS Battista V Oliverio JA. Safety and efficacy of intraarticular hip injection using anatomic landmarks. Clin Orthop Relat Res®. (2001) 391:192. 10.1097/00003086-200110000-00021 11603669

[8] Reilly I Chockalingam N Naemi R. The accuracy of first metatarsophalangeal joint palpation guided injections. An arthrography cadaveric study. Foot & ankle surgery: techniques. Rep Cases. (2022) 2(3):100219. 10.1016/j.fastrc.2022.100219

[9] Heidari N Kraus T Fischerauer S Tesch N Weinberg A. Do the presence of pathologic changes and the level of operator experience alter the rate of intra-articular injection of the first metatarsophalangeal joint? A cadaver study. J Am Podiatr Med Assoc. (2013) 103(3):204–7. 10.7547/1030204 23697725

[10] Hagemeijer NC Lubberts B Saengsin J Bhimani R Sato G Waryasz GR Portable dynamic ultrasonography is a useful tool for the evaluation of suspected syndesmotic instability: a cadaveric study. Knee Surg Sports Traumatol Arthrosc. (2023) 31(5):1986–93. 10.1007/s00167-022-07058-4 35881148 PMC10089982

[11] Anderson SE Lubberts B Strong AD Guss D Johnson AH DiGiovanni CW. Adverse events and their risk factors following intra-articular corticosteroid injections of the ankle or subtalar joint. Foot Ankle Int. (2019) 40(6):622–8. 10.1177/1071100719835759 30866653

[12] Gonçalves B Ambrosio C Serra S Alves F Gil-Agostinho A Caseiro-Alves F. US-guided interventional joint procedures in patients with rheumatic diseases—when and how we do it? Eur J Radiol. (2011) 79(3):407–14. 10.1016/j.ejrad.2010.04.001 20554144

[13] Raza K Lee CY Pilling D Heaton S Situnayake RD Carruthers DM Ultrasound guidance allows accurate needle placement and aspiration from small joints in patients with early inflammatory arthritis. Rheumatology. (2003) 42(8):976–9. 10.1093/rheumatology/keg269 12730511

[14] Reach JS Easley ME Chuckpaiwong B Nunley JA. Accuracy of ultrasound guided injections in the foot and ankle. Foot Ankle Int. (2009) 30(3):239–42. 10.3113/FAI.2009.0239 19321101

[15] Smith J Finnoff JT Levy BA Lai JK. Sonographically guided proximal tibiofibular joint injection. J Ultrasound Med. (2010) 29(5):783–9. 10.7863/jum.2010.29.5.783 20427791

[16] Wempe MK Sellon JL Sayeed YA Smith J. Feasibility of first metatarsophalangeal joint injections for sesamoid disorders: a cadaveric investigation. PM R. (2012) 4(8):556–60. 10.1016/j.pmrj.2012.01.011 22484333

[17] Wisniewski SJ Smith J Patterson DG Carmichael SW Pawlina W. Ultrasound-guided versus nonguided tibiotalar joint and Sinus tarsi injections: a cadaveric study. PM R. (2010) 2(4):277–81. 10.1016/j.pmrj.2010.03.013 20430329

[18] Jha AJ Viner GC McKissack H Anderson M Prather J Shah AB Accuracy of talonavicular injection using ultrasound versus anatomical landmark: a cadaver study. Acta Radiol. (2020) 61(10):1359–64. 10.1177/0284185120901507 32008342

[19] Emami Razavi SZ Azadvari M Fateh HR Ghahvechi Akbari M Kazemi S Rezaee E. Short-term efficacy of ultrasonographic guidance for intra-articular corticosteroid injection in hallux rigidus: a single-blind randomized controlled trial. Foot Ankle Int. (2021) 42(11):1410–8. 10.1177/10711007211015988 34111992

[20] Lucas PE Hurwitz SR Kaplan PA Dussault RG Maurer EJ. Fluoroscopically guided injections into the foot and ankle: localization of the source of pain as a guide to treatment–prospective study. Radiology. (1997) 204(2):411–5. 10.1148/radiology.204.2.9240528 9240528

[21] Patel A Chadwick N von Beck K Goswami P Soliman SB Patel A Ultrasound-guided joint interventions of the lower extremity. Skeletal Radiol. (2023) 52(5):911–21. 10.1007/s00256-022-04168-5 36042035

[22] Ruhoy MK Newberg AH Yodlowski ML Mizel MS Trepman E. Subtalar joint arthrography. Semin Musculoskelet Radiol. (1998) 2(04):433–7. 10.1055/s-2008-1080123 11387121

[23] Sibbitt WL Band PA Chavez-Chiang NR DeLEA SL Norton HE Bankhurst AD. A randomized controlled trial of the cost-effectiveness of ultrasound-guided intraarticular injection of inflammatory arthritis. J Rheumatol. (2011) 38(2):252–63. 10.3899/jrheum.100866 21078710

[24] Drakonaki EE Kho JSB Sharp RJ Ostlere SJ. Efficacy of ultrasound-guided steroid injections for pain management of midfoot joint degenerative disease. Skeletal Radiol. (2011) 40(8):1001–6. 10.1007/s00256-010-1094-y 21274710

